# Consistent changes in the intestinal microbiota of Atlantic salmon fed insect meal diets

**DOI:** 10.1186/s42523-021-00159-4

**Published:** 2022-01-10

**Authors:** Yanxian Li, Karina Gajardo, Alexander Jaramillo-Torres, Trond M. Kortner, Åshild Krogdahl

**Affiliations:** Department of Paraclinical Sciences, Faculty of Veterinary Medicine, Norwegian University of Life Sciences, Oslo, Norway

**Keywords:** Atlantic salmon, Insect meal, Black soldier fly, Intestinal microbiota, Feed microbiota, Water microbiota

## Abstract

**Background:**

Being part of fish's natural diets, insects have become a practical alternative feed ingredient for aquaculture. While nutritional values of insects have been extensively studied in various fish species, their impact on the fish microbiota remains to be fully explored. In an 8-week freshwater feeding trial, Atlantic salmon (*Salmo salar*) were fed either a commercially relevant reference diet or an insect meal diet wherein black soldier fly (*Hermetia illucens*) larvae meal comprised 60% of total ingredients. Microbiota of digesta and mucosa origin from the proximal and distal intestine were collected and profiled along with feed and water samples.

**Results:**

The insect meal diet markedly modulated the salmon intestinal microbiota. Salmon fed the insect meal diet showed similar or lower alpha-diversity indices in the digesta but higher alpha-diversity indices in the mucosa. A group of bacterial genera, dominated by members of the *Bacillaceae* family, was enriched in salmon fed the insect meal diet, which confirms our previous findings in a seawater feeding trial. We also found that microbiota in the intestine closely resembled that of the feeds but was distinct from the water microbiota. Notably, bacterial genera associated with the diet effects were also present in the feeds.

**Conclusions:**

We conclude that salmon fed the insect meal diets show consistent changes in the intestinal microbiota. The next challenge is to evaluate the extent to which these alterations are attributable to feed microbiota and dietary nutrients, and what these changes mean for fish physiology and health.

**Supplementary Information:**

The online version contains supplementary material available at 10.1186/s42523-021-00159-4.

## Background

The global population is projected to reach 9.7 billion in 2050 [1], requiring an increase in the food supply by 25–70% [2]. To fulfil this demand, the food production sector must minimize resource input and maximize nutritional outputs for human consumption. Atlantic salmon, *Salmo salar*, is the most produced marine fish species and one of the most economically important farmed fish worldwide [3]. Human-edible plant feedstuffs are the main ingredients used in modern salmon feeds (~ 70%) [4]. To secure sustainable developments, salmon farming needs to decrease its dependency on human-edible feedstuffs and incorporate unexploited feed resources in its raw material repertoire. So far, possible candidates include insects [5], macroalgae [6], and single-cell organisms such as bacteria, yeasts, and microalgae [7]. In terms of sustainability, insects are a promising candidate. They possess a remarkable capacity to upgrade low-quality organic materials, require minimal water and cultivable land, and emit little greenhouse gases [8]. One of the insect species with the potential as alternative protein sources for salmon aquaculture is black soldier fly (*Hermetia illucens*), which is produced at an industrial scale for its favorable amino acid profile [9]. Feed conversion ratio, growth performance, fish health, sustainability and price/availability are primary concerns when evaluating the performance of alternative feed ingredients. While the nutritional value of black soldier fly larvae meal has been extensively evaluated in various fish species, including Atlantic salmon [10–16], its influence on fish health remains largely unexplored.

The intestine is the main organ directly exposed to the diet and of pivotal importance for the growth, development, and protection against pathogens. A well-functioning, healthy intestine is the key to convert feed into fish biomass efficiently. It is now well established that the intestinal microbiota is, in various ways, closely connected to intestinal function and health [17–21]. Diet is arguably one of the most important environmental factors shaping intestinal microbiota [22–24]. Different dietary components may selectively induce compositional and functional alterations of the intestinal microbiota, which in turn could inflict important implications on the host health and disease resistance [19, 24–26].

Characterizing the response of intestinal microbiota to dietary shifts and its associations with host responses is a critical step towards identifying key microbial clades for promoting fish health and welfare. The main aims of the work presented herein were (1) to compare intestinal microbiota of Atlantic salmon fed a commercially relevant reference diet and an insect meal-based test diet, and (2) to identify potential associations between intestinal microbial clades and host responses. This work was part of a larger study consisting of a freshwater and a seawater feeding trial. The present work reports the intestinal microbiota in freshwater Atlantic salmon fed an insect meal diet containing 60% black soldier fly larvae meal for 8 weeks.

## Results

Published results on the growth performance, intestinal histomorphology, and gene expression are summarized as the following [27, 28]. In brief, there was little evidence that the insect meal diet negatively affected salmon's feed utilization or growth performance. Histopathological examination showed excessive accumulation of lipids (steatosis) in the proximal intestine in both diet groups, but it was less severe in salmon fed the insect meal diet. The expression of the lipid droplet marker gene, *plin2*, supported these histological findings. Immune and barrier-function gene expression profiles were generally not affected by diet. However, salmon fed the insect meal diet showed increased expression of genes indicative of immune tolerance (*foxp3*), stress response (*hsp70*), and detoxification activity (*cpy1a1*).

### Taxonomic analysis

**Fig. 1:**
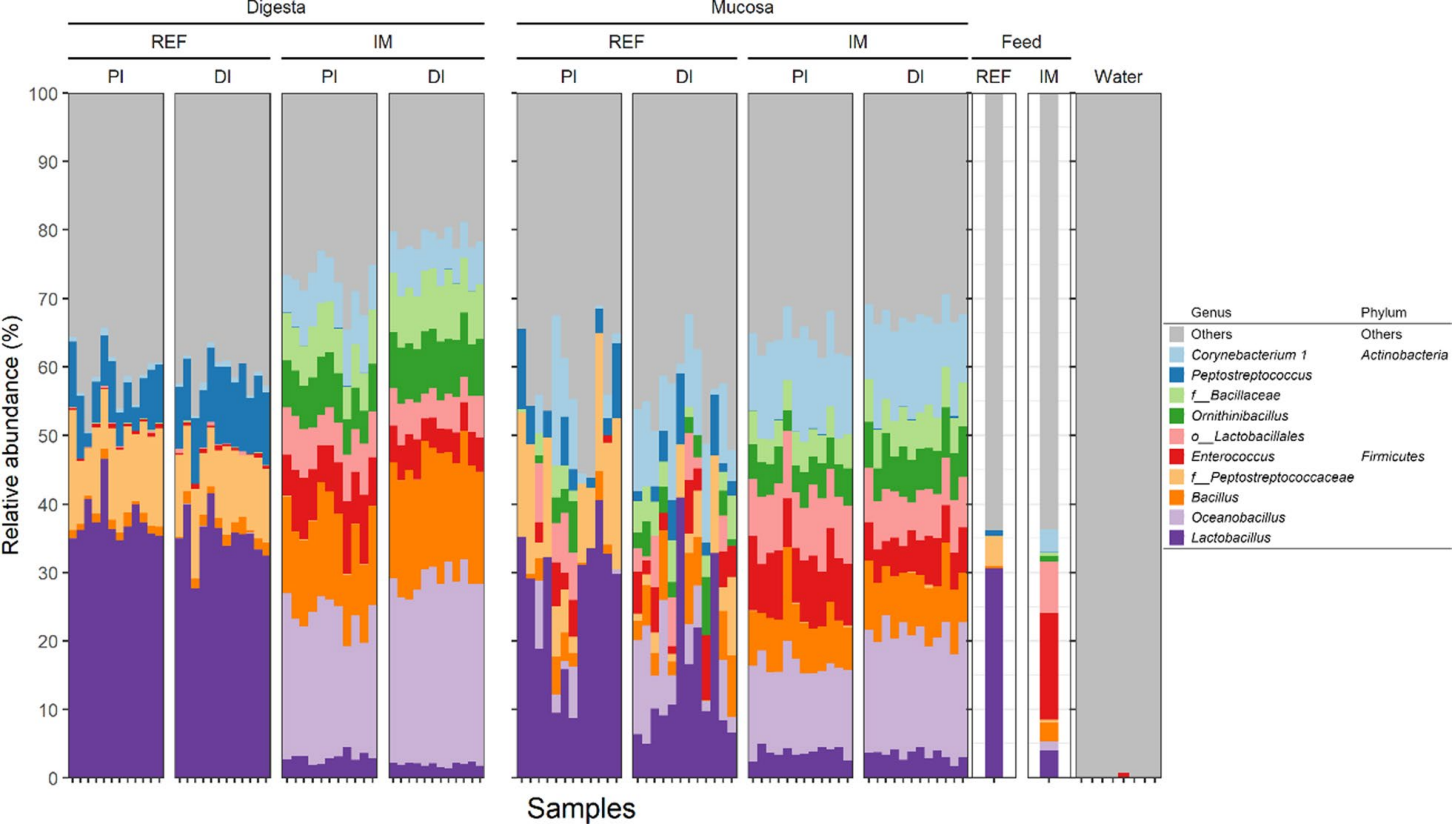
Consistent changes in the taxonomic composition of intestinal microbiota from salmon fed the insect meal diet. Note that feed microbiota shows close resemblance to that observed in the intestine whereas water microbiota is very distinct from the intestinal microbiota. Only the top 10 most abundant bacterial genera are displayed in the plot whereas the other taxa are shown as “Others”. Taxa not assigned at the genus level are prepended with letters indicating whether the taxonomic assignment was made at the order (o_) or family (f_) level. Abbreviations: REF, reference diet; IM, insect meal diet; PI, proximal intestine; DI, distal intestine

**Fig. 2:**
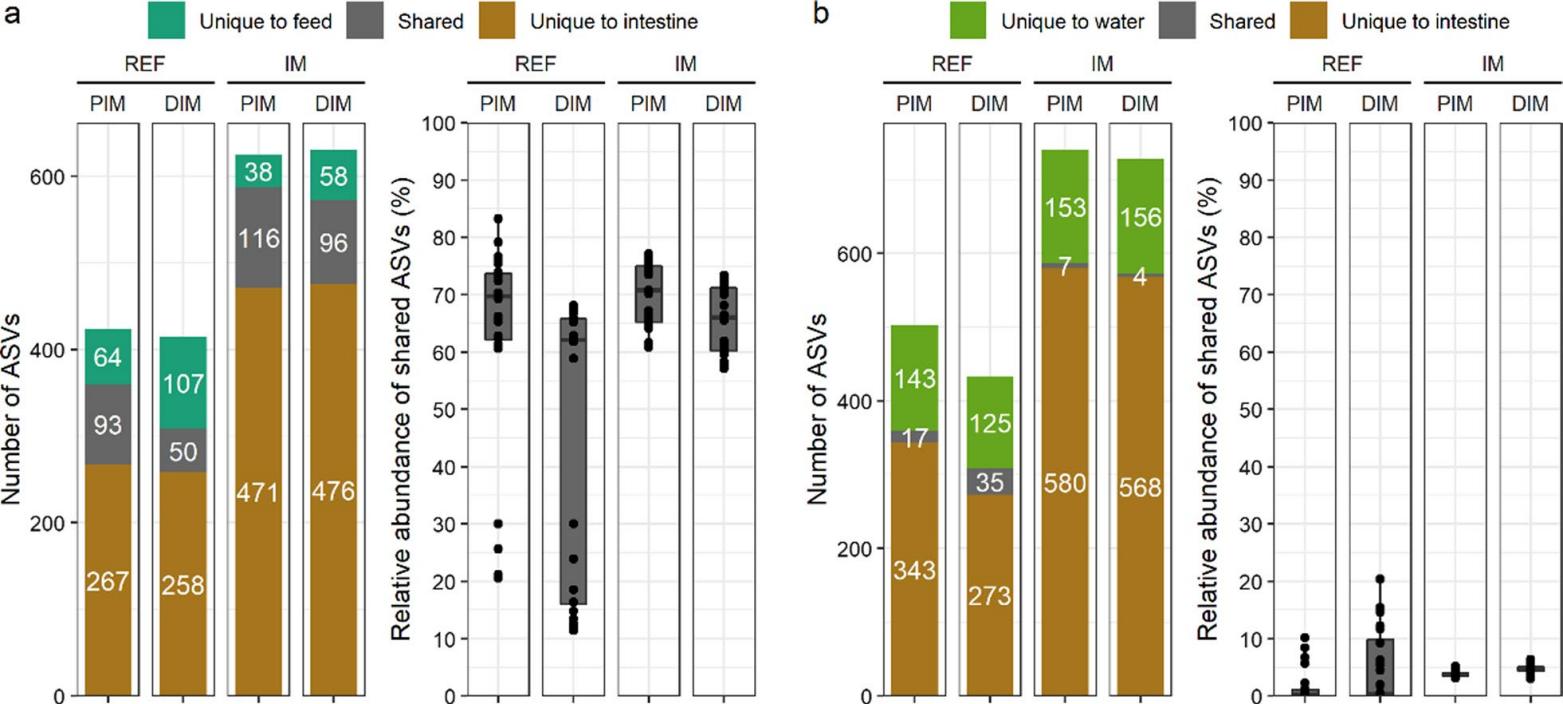
Higher microbial overlap between the intestinal mucosa and feeds (**a**) than that between the intestinal mucosa and water (**b**). In each panel, the number of shared ASVs is shown on the left whereas the relative abundance of shared ASVs in the intestinal mucosa is shown on the right. To reduce the influence of rare ASVs and differences in the sequencing depth, only ASVs with a minimum relative abundance of 0.05% were considered as present in a sample. Abbreviations: REF, reference diet; IM, insect meal diet; PIM, proximal intestine mucosa; DIM, distal intestine mucosa

The observed taxonomic composition of the mock standard is shown in Additional file 2: Figure S1. Contaminants identified in the negative control samples are shown in Additional file 1: Table S1. The top 10 most abundant bacterial genera across all the samples are shown in Fig. 1. At visual observation, the microbiota in the digesta collected from the two intestinal segments of the salmon fed the reference diet appeared homogenous, but more heterogeneous in the sampled mucosa. Dominant genera in the reference diet group included *Lactobacillus*, unclassified *Peptostreptococcaceae,* and *Peptostreptococcus*. The microbiota in salmon fed the insect meal diet differed greatly from that of the reference diet fed fish, and the difference between the results of the digesta and mucosa appeared less than for fish fed the reference diet. Dominant genera in the insect meal diet group included *Oceanobacillus*, *Bacillus*, *Enterococcus*, *Ornithinibacillus*, unclassified *Bacillaceae*, and *Corynebacterium 1*. The microbiota in the intestine closely resembled that of the feed but was distinct from the water microbiota. In agreement with this, we found that the ASV overlap between the intestine and feed was much higher than that between the intestine and water (Fig. 2).

### Alpha-diversity

**Fig. 3:**
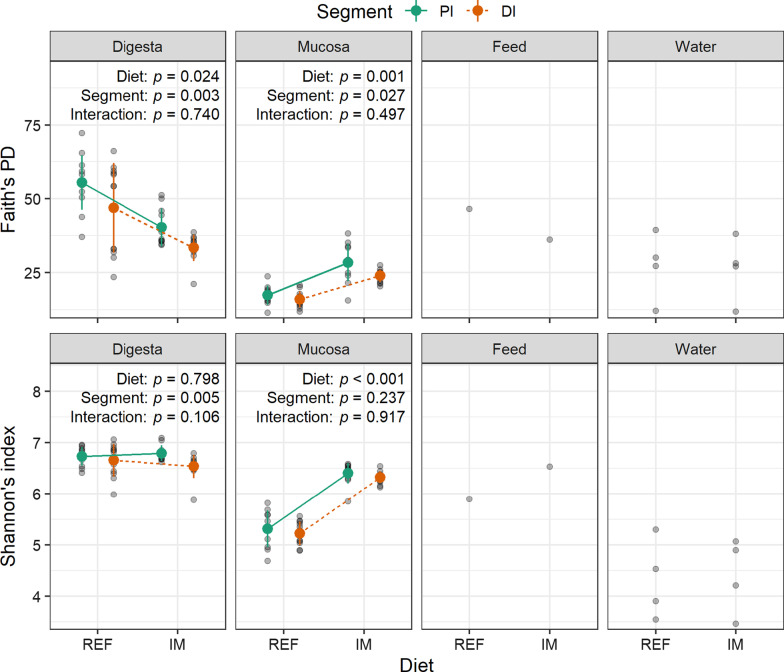
Salmon fed the insect meal diet showed similar or lower alpha-diversity indices in the digesta but higher alpha-diversity indices in the mucosa. The error bars denote standard deviations of the means. The *p* values of the main effects and their interaction are displayed on the top of each subplot. Abbreviations: REF, reference diet; IM, insect meal diet; PI, proximal intestine; DI, distal intestine; PD, phylogenetic diversity

Salmon fed the insect meal diet showed similar or lower alpha-diversity indices in the digesta but higher alpha-diversity indices in the mucosa (Fig. 3). In the digesta, regardless of the intestinal segment, salmon fed the insect meal diet showed significantly lower Faith’s phylogenetic diversity but similar Shannon’s index. In the mucosa, however, the Faith’s phylogenetic diversity and Shannon’s index were significantly higher in salmon fed the insect meal diet in both intestinal segments.

Compared with the intestinal mucosal samples, the water samples showed higher, albeit not significant, Faith’s phylogenetic diversity but significantly lower Shannon’s index (Additional file 2: Figure S2).

### Beta-diversity

**Fig. 4:**
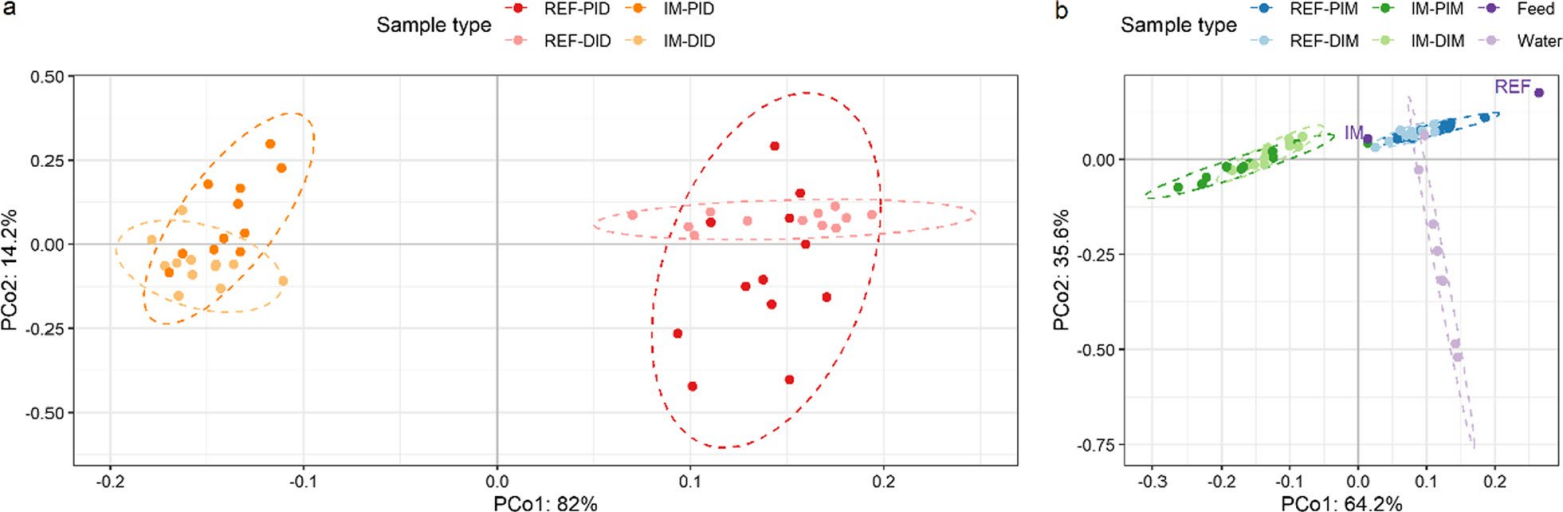
The insect meal diet markedly modulated the salmon intestinal microbiota in both digesta (**a**) and mucosa (**b**), irrespective of intestinal segments. The dimensionality reduction was performed using a compositional beta-diversity metric called robust Aitchison PCA and visualized by the EMPeror [90]. The height-to-width ratio of the PCoA plot was set to reflect the ratio between the corresponding eigenvalues as recommended [91]. Abbreviations: REF, reference diet; IM, insect meal diet; PID, proximal intestine digesta; DID, distal intestine digesta; PIM, proximal intestine mucosa; DIM, distal intestine mucosa; PCo, principal coordinate

In the digesta, the PERMANOVA showed a significant diet but not a significant intestinal segment effect on the beta-diversity, and the interaction between these terms was significant (Fig. 4a; Table 1). The diet effect on the beta-diversity was significant in both intestinal segments, but it was stronger in the distal intestine than in the proximal intestine. The PERMDISP showed that, in both intestinal segments, differences in the multivariate dispersion between the diet groups were not significant (Additional file 2: Figure S3a).

**Table 1:** PERMANOVA and subsequent conditional contrasts

Source	Main effects		Interaction	Conditional contrasts			
	Diet	Segment		REF-PI versus IM-PI	REF-DI versus IM-DI	REF-PI versus REF-DI	IM-PI versus IM-DI
Digesta	*F* = 50.5, *p* = 0.001	*F* = 0.481, *p* = 0.503	*F* = 16.1, *p* = 0.002	*t* = 4.30, *p* = 0.001	*t* = 10.7, *p* = 0.001	NA	NA
Mucosa	*p* = 0.001^a^	*F* = 0.449, *p* = 0.591	*F* = 6.04, *p* = 0.059	NA	NA	NA	NA

REF, reference diet; IM, insect meal diet; PI, proximal intestine; DI, distal intestine; NA, not applicable

^a^Monte Carlo *p *value

In the mucosa, the PERMANOVA showed a significant diet but not a significant intestinal segment effect on the beta-diversity, and the interaction between these terms was not significant (Fig. 4b; Table 1). The PERMDISP showed that differences in the multivariate dispersion between the diet groups were not significant at the tank or diet level (Additional file 2: Figure S3b).

The PERMANOVA showed that the water microbiota was significantly different from the intestinal mucosal microbiota (*p* = 0.003). Differences in the multivariate dispersion (PERMDISP) between the water and intestinal mucosal samples were not significant (*p* = 0.140).

### Association analysis

**Fig. 5:**
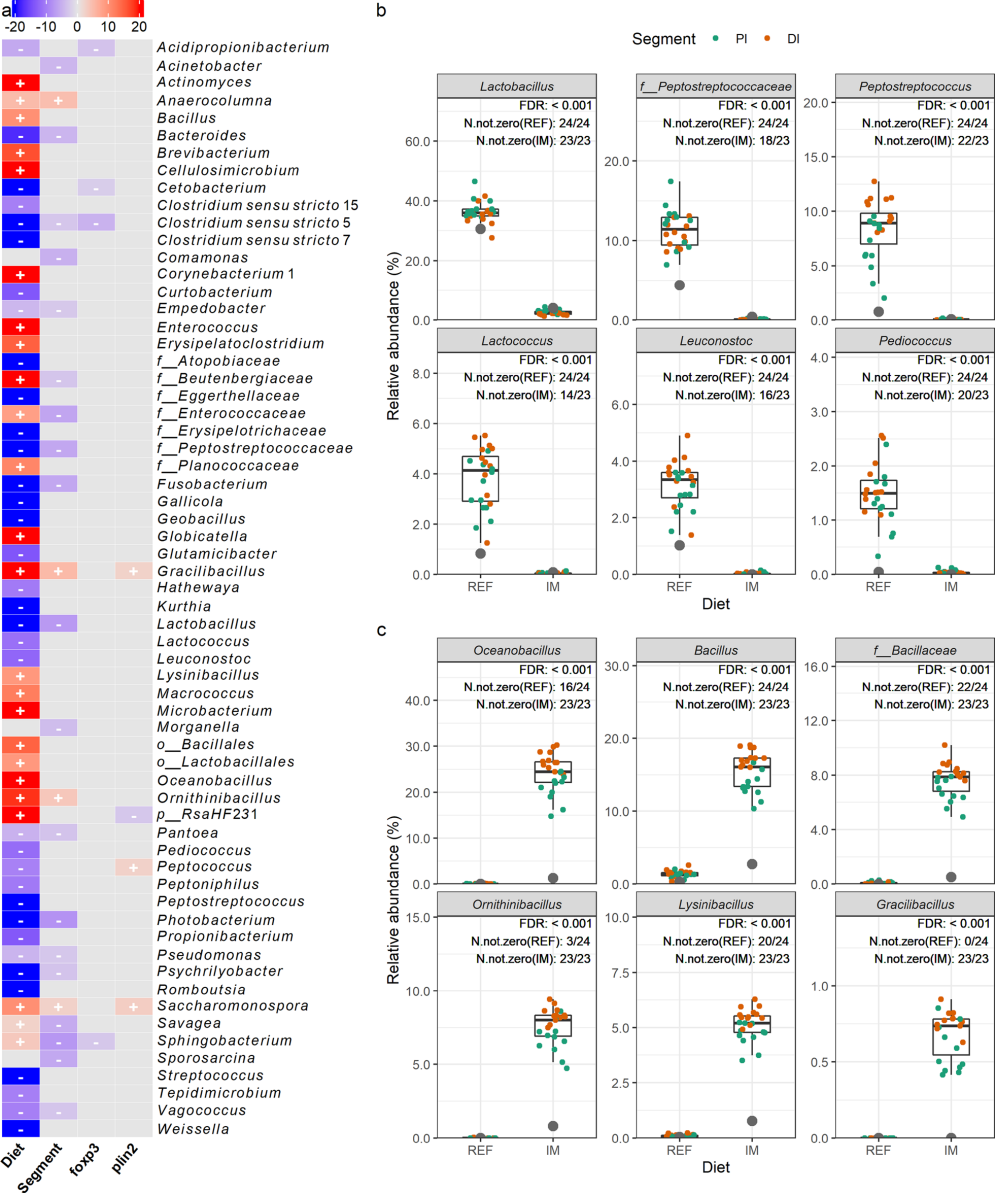
Significant associations between sample metadata and microbial clades in the digesta. **a** Heatmap summarizing significant associations between sample metadata and microbial clades in the digesta. Color key: − log(*q *value) * sign(coefficient). Cells that denote significant associations are colored in red or blue and overlaid with a plus (+) or minus (−) sign that indicates the direction of association: Diet (+), higher relative abundance in salmon fed the insect meal diet; Segment (+), higher relative abundance in the distal intestine; *foxp3* (+)/*plin2* (+), positive correlation between microbial clade relative abundance and gene expression levels. **b** Representative taxa showing higher relative abundances in salmon fed the reference diet. **c** Representative taxa showing higher relative abundances in salmon fed the insect meal diet. The relative abundances of representative taxa in the feeds are shown as grey dots in **b**, **c**. As the number of taxa showing significant associations with diet was too high to be properly displayed on the heatmap, we filtered the results to keep those with a *q* value < 0.0001. Complete results are available in our accompanying R Markdown report (download our GitHub repository, https://github.com/yanxianl/Li_AqFl1-Microbiota_2021, and open the file code/11_multivariable_association.html). Taxa not assigned at the genus level are prepended with letters indicating whether the taxonomic assignment was made at the phylum(p_), order (o_), or family (f_) level. REF, reference diet; IM, insect meal diet; PI, proximal intestine; DI, distal intestine; FDR, false discovery rate; N.not.zero, number of observations that are not zero

**Fig. 6:**
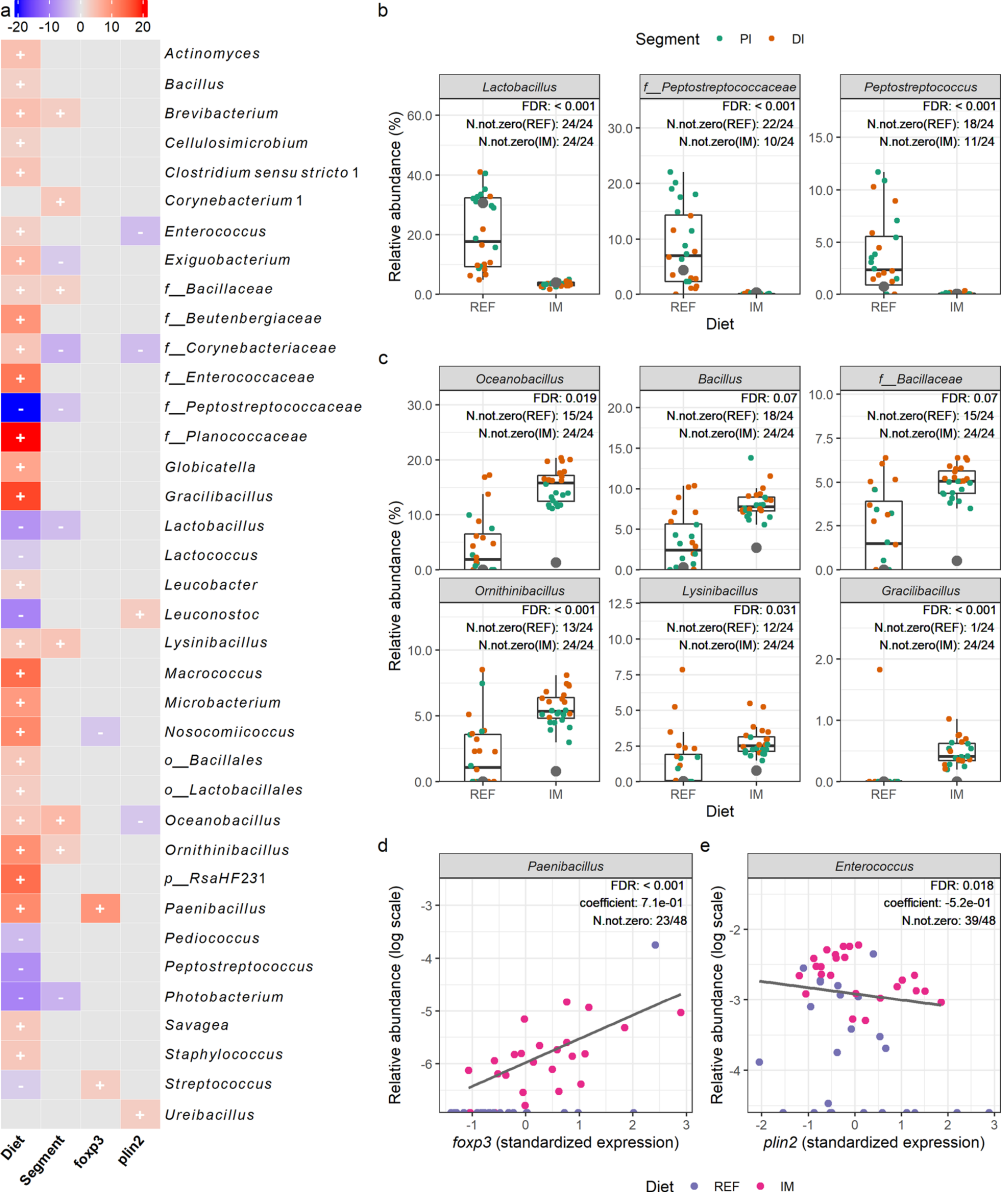
Significant associations between sample metadata and microbial clades in the mucosa. **a** Heatmap summarizing significant associations between sample metadata and microbial clades in the mucosa. Color key: − log(*q *value) *sign(coefficient). Cells that denote significant associations are colored in red or blue and overlaid with a plus (+) or minus (−) sign that indicates the direction of association: Diet (+), higher relative abundance in salmon fed the insect meal diet; Segment (+), higher relative abundance in the distal intestine; *foxp3* (+)/*plin2* (+), positive correlation between microbial clade relative abundance and gene expression levels. **b** Representative taxa showing higher relative abundances in salmon fed the reference diet. **c** Representative taxa showing higher relative abundances in salmon fed the insect meal diet. **d** Positive correlation between the relative abundance of *Paenibacillus* and *foxp3* expression levels in the intestine. **e** Negative correlation between the relative abundance of *Enterococcus* and *plin2* expression levels in the intestine. The relative abundances of representative taxa in the feeds are shown as grey dots in **b**, **c**. Taxa not assigned at the genus level are prepended with letters indicating whether the taxonomic assignment was made at the phylum(p_), order (o_), or family (f_) level. REF, reference diet; IM, insect meal diet; PI, proximal intestine; DI, distal intestine; FDR, false discovery rate; N.not.zero, number of observations that are not zero

Significant associations between sample metadata and bacterial genera in the digesta and mucosa are shown in Figs. 5 and 6, respectively. In total, 89 and 35 taxa were associated with the diet effect in the digesta and mucosa, respectively. Collectively, 32 taxa were associated with the diet effect in both digesta and mucosa. Among these taxa, bacterial genera enriched in salmon fed the reference diet consisted of unclassified *Peptostreptococcaceae*, *Peptostreptococcus*, *Photobacterium,* and lactic acid bacteria including *Lactobacillus*, *Lactococcus*, *Leuconostoc*, *Pediococcus,* and *Streptococcus* (partially illustrated in Figs. 5b, 6b). In contrast, bacterial genera enriched in salmon fed the insect meal diet comprised *Actinomyces*, unclassified *Bacillales*, unclassified *Bacillaceae*, *Bacillus*, unclassified *Beutenbergiaceae*, *Brevibacterium*, *Cellulosimicrobium*, *Clostridium *sensu stricto* 1*, unclassified *Corynebacteriaceae*, unclassified *Enterococcaceae*, *Enterococcus*, *Exiguobacterium*, *Globicatella*, *Gracilibacillus*, unclassified *Lactobacillales*, *Lysinibacillus*, *Macrococcus*, *Microbacterium*, *Oceanobacillus*, *Ornithinibacillus*, *Paenibacillus*, unclassified *Planococcaceae*, unclassified *RsaHF231* and *Savagea* (partially illustrated in Figs. 5c, 6c). Regarding associations between bacterial genera and host gene expressions, the relative abundance of *Paenibacillus* and *Streptococcus* in the mucosa showed positive correlations with the expression level of *foxp3*, the master transcription factor of regulatory T-cells, in the intestine (partially illustrated in Fig. 6d). Additionally, the relative abundance of unclassified *RsaHF231* in the digesta, and the relative abundance of unclassified *Corynebacteriaceae*, *Enterococcus,* and *Oceanobacillus* in the mucosa, showed negative correlations with the expression level of *plin2*, a surface marker of lipid droplets, in the intestine (partially illustrated in Fig. 6e).

## Discussion

We found that the insect meal diet markedly modulated the Atlantic salmon intestinal microbiota. A group of bacterial genera, dominated by members of the *Bacillaceae* family, was enriched in salmon fed the insect meal diet. These results confirm our previous findings in a seawater feeding trial [29]. We also found that microbiota in the intestine closely resembled that of the feeds. Notably, bacterial genera associated with the diet effects were present in the feeds as well.

### Insect meal diet markedly modulated the intestinal microbiota

Higher microbial diversity has been reported in the intestinal digesta and mucosa of salmonids fed diets containing black soldier fly larvae meal [29–32]. In the present study, however, this was the case for the mucosa but not for the digesta. Our observation that a particular group of bacterial genera, dominated by members of the *Bacillaceae* family, was enriched in salmon fed the insect meal diet is in line with findings in our previous seawater trial, wherein salmon were fed an insect meal diet containing 15% black soldier fly larvae meal for 16 weeks [29]. Among these bacterial genera, *Actinomyces*, *Bacillus*, *Brevibacterium*, *Corynebacterium 1, Enterococcus*, *Oceanobacillus*, and *Paenibacillus* were also reported to be enriched in rainbow trout fed diets containing 15% or 30% black soldier fly larvae meal [31–33]. Similar observations have been made in Siberian sturgeon (*Acipenser baerii*) fed a diet containing 15% black soldier fly larvae meal, inducing higher absolute abundances of *Bacillus* and *Enterococcus* [34]. In this latter study, fluorescence in situ hybridization (*FISH*) technique was used for the bacteria quantification.

Feed microbiota and dietary nutrients may explain the observed diet effects. We found evidence for the former, because bacterial genera associated with the diet effects were present in the feed samples. Given the hydrothermal treatments during the extrusion step in the feed production, the viability of feed-associated microbes is expected to be low. As sequencing-based methods cannot differentiate between active (living) and inactive (dormant/dead) microbes, additional work will be needed to elucidate the extent to which the observed diet effects are attributable to the carry-over of inactive microbes and colonization of active microbes from feeds. Methods like viability PCR and RNA sequencing can be applied for such experiments [35]. Changes in the feed components may have also contributed to the observed diet effects. For instance, dietary inclusion of soy proteins was suggested to associate with increased relative abundance of lactic acid bacteria in the salmon intestine [36]. Thus, the replacement of soy protein concentrate with insect meal may explain the reduction in lactic acid bacteria in salmon fed the insect meal diet. On the other hand, nutrients from the insect meal, such as chitin, may have also promoted the growth of certain bacterial taxa including *Actinomyces* and *Bacillus*. *Actinomyces* species are often identified as active chitin degraders, showing enhanced growth and activity upon chitin addition [37]. Many *Bacillus* species are well-known as chitin degraders [38]. *Bacillus* was one of the predominant taxa in the intestinal mucosa of salmon fed a chitin-supplemented diet, displaying the highest in vitro chitinase activity [39]. The latter hypothesis can be tested by supplementing insect meal-specific nutrients to the same basal diet and sequencing the intestinal microbiota of salmon fed these diets.

### Microbiota was similar between intestinal segments

Like its mammalian counterparts [40, 41], the salmon intestinal microbiota is also spatially heterogeneous in its composition [42]. Specifically, microbial communities differ along the intestinal tract and vary substantially between digesta and mucosa within the same intestinal segment. Due to the batch effects between sequencing runs, we could not directly compare microbial communities in the digesta and mucosa. Nonetheless, our study suggests that conclusions on the diet effect can be different when evaluated using digesta or mucosa samples alone. This is supported by our results showing that diet effects on the alpha-diversity and differential abundance testing were quite different when evaluated independently using digesta or mucosa samples. In contrast, our comparative analysis showed that microbiota variations between intestinal segments were neglectable in both digesta and mucosa. The diet effects were essentially the same when evaluated using samples from different intestinal segments. Taken together, these results suggest that it may be sufficient to collect digesta and mucosa samples from one intestinal segment (e.g., the distal intestine) when conducting a diet-microbiota study in fish with limited resources.

### Microbial overlap was low between the intestine and water but high between the intestine and feeds

Water and feed are considered two environmental sources of microbiota which can be transferred to the fish intestine. In line with previous studies in salmon [43–45] and other fish species [46–48], we found that microbial overlap between the intestine and water was low in the present study of salmon in freshwater. This may be explained by the fact that during their freshwater stage, salmon drink little water to accommodate osmoregulation needs in a hypo-osmotic environment, which greatly limits the intake of microbes from the surrounding water environment. On the other hand, the microbial load in the tank water may be low as it was heavily filtered before supplied to the fish rearing system. The tank biofilm may be a better indicator of environmental microbiota [49] as it enriches microbes that naturally colonize the system. Sequencing both water and tank biofilm microbiota may provide better insights into interactions between the environmental microbiota and fish intestinal microbiota.

In contrast to the low microbial overlap between the intestine and water, we found a high microbial overlap between the intestine and feed. Microbial overlaps between the fish intestine and formulated feeds have been reported to be high [50, 51] and low [52–55] in the literature. As discussed earlier, the feed microbiota detected by amplicon sequencing may have primarily originated from inactive microbes. Therefore, feed microbiota can be a confounding factor of the observed diet effects. Given that the influence of feed microbiota on the observed diet effects is unequal across experimental groups as opposed to the water microbiota, we strongly recommend collecting feed samples when designing a sequencing-based, diet-microbiota study in fish.

### Associations between microbial clades and host gene expressions

The close relationship between microbiota and the intestinal immune system is well established [56]. *Paenibacillus* are endospore-forming, facultative anaerobes well known as plant-growth promoters [57]. Metabolites produced by *Paenibacillus* have been reported to downregulate inflammatory response and increase regulatory T cell numbers in the intestine of Goto–Kakizaki rats, a spontaneous animal model of type 2 diabetes [58]. In accordance, we found that *Paenibacillus* was positively associated with the *foxp3* expression, suggesting a putative link between the enrichment of *Paenibacillus* and increased expression of *foxp3* in salmon fed the insect meal diet. Interaction between microbiota and lipid metabolism in the intestine has also been documented [59, 60]. Intestinal steatosis is a condition caused by excessive lipid accumulation within enterocytes. It represents a lipid transport disorder likely caused by deficiencies in nutrients required for the lipoprotein assembly [61–63]. Our findings, showing that several bacterial clades enriched in salmon fed the insect meal diet were negatively associated with the expression of lipid droplet marker *plin2*, indicate that the intestinal microbiota might also play a role in the development of intestinal steatosis. However, as microbiome data are sparse and noisy, association analysis is more meaningful when the sample size is much larger than it was in this study. Given the limited sample size, our results should be interpreted as exploratory. Further research is required to test if these bacterial taxa are indeed involved in the immune modulation and lipid metabolism in the salmon intestine.

## Conclusions

Our work showed that the insect meal diet markedly modulated the Atlantic salmon intestinal microbiota. Salmon fed the insect meal diet showed similar or lower alpha-diversity indices in the digesta but higher alpha-diversity indices in the mucosa. A group of bacterial genera, dominated by members of the *Bacillaceae* family, was enriched in salmon fed the insect meal diet. These results support our previous findings from a study of Atlantic salmon in seawater. We also found that microbiota in the intestine closely resembled that of the feed but was distinct from the water microbiota. Notably, bacterial genera associated with the diet effects were present in the feed samples as well. We conclude that salmon fed the insect meal diets show consistent changes in the intestinal microbiota. The next challenge is to evaluate the extent to which these alterations are attributable to feed microbiota and dietary nutrients, and what these changes mean for fish physiology and health.

## Methods

### Diet and fish husbandry

An 8-week freshwater feeding trial was conducted at Cargill AquaNutrition experimental facility at Dirdal, Norway. A total of 800 Atlantic salmon with a mean initial body weight of 49 g (1.5 g SEM) were randomly assigned into 8 fiberglass tanks (450 L, 100 fish per tank) supplied with running freshwater. Quadruplicate tanks of fish were fed either a reference diet with a combination of fish meal, soy protein concentrate, and wheat gluten as protein sources, or an insect meal diet wherein 85% of the protein was supplied by black soldier fly larvae meal, replacing most of the fish meal and soy protein concentrate (Table 2). The black soldier fly larvae were grown on feed substrates containing organic waste streams. After eight days of growing, the larvae were harvested and partially defatted before being dried and ground to make the insect meal (Protix Biosystems BV, Dongen, The Netherlands). The diets were extruded, dried and vacuum coated with oils, producing feed pellets with a diameter size of 2 mm (Cargill, Dirdal, Norway). After the production, the diets were shipped to the experimental facility and stored at − 20 °C until use. The fish were fed continuously by automatic disk feeders under a photoperiod regimen of 24 h daylight. Uneaten feeds were collected from tank outlets and registered daily. During the feeding trial, the water temperature was 13.7 ± 0.1 °C, and the dissolved oxygen concentration of the inlet and outlet water was 11.9 ± 1.2 and 8.7 ± 0.5 mg/L, respectively. Further details on the nutritional composition of the insect meal and diets have been reported elsewhere [28, 64].

**Table 2:** Formulation of the experimental diets

Ingredients (g/100 g)	REF	IM
Fishmeal LT94	35.0	6.0
Insect meal	0	60.0
Soy protein concentrate	29.6	5.0
Wheat gluten	14.3	14.4
Fish oil	4.6	6.9
Rapeseed oil	12.0	4.8
Vitamin and mineral premix	0.3	0.3
Yttrium	0.2	0.2
Miscellaneous	4.0	2.4
*Chemical composition*		
Dry matter (%)	94	96
Crude lipid (%)	18	22
Crude protein (%)	47	44
Carbohydrates (%)	11	12
Ash (%)	8	7
Gross energy (MJ/Kg dry matter)	22	23
TBARS (nmol/g)	7	17

REF, reference diet; IM, insect meal diet; TBARS, Thiobarbituric acid reactive substances

### Sample collection

At the termination of the feeding trial, 3 fish were randomly taken from each tank (i.e., 12 fish per treatment), anesthetized with tricaine methanesulfonate (MS222®; Argent Chemical Laboratories, Redmond, WA, USA), and euthanized by a sharp blow to the head. After cleaning the exterior of each fish with 70% ethanol, the proximal and distal intestine were aseptically removed from the abdominal cavity, placed in sterile Petri dishes, and opened longitudinally. Only fish with digesta along the whole intestine were sampled to ensure that the intestine had been exposed to the diets. The intestinal digesta was gently removed and transferred into a 1.5 mL sterile Eppendorf tube using a spatula and snap-frozen in liquid N_2_ for the profiling of digesta-associated intestinal microbiota. The intestinal tissue was rinsed in sterile phosphate-buffered saline 3 times to remove traces of remaining digesta. After rinsing, the intestinal tissue was cut into 3 pieces for histological evaluation (fixed in 4% phosphate-buffered formaldehyde solution for 24 h and transferred to 70% ethanol for storage), gene expression analysis (preserved in RNAlater solution and stored at − 20 °C), and profiling of mucosa-associated intestinal microbiota (snap-frozen in liquid N_2_), respectively. In addition, 300 mL water was taken from each tank, pre-filtered through a 0.8 μm sterile syringe filter (Acrodisc®, Pall Corporation, New York, USA), and vacuum-filtered onto a 0.2 μm sterile nitrocellulose filter (Nalgene™, Thermo Scientific, USA). The filter containing enriched bacteria was folded, placed into an 8 mL sterile tube, and snap-frozen in liquid N_2_ to profile microbial community in water. The collection of microbiota samples was performed near a gas burner to secure aseptic conditions. Tools were cleaned and decontaminated by 70% ethanol sprays and flaming before the subsequent sampling was carried out. The samples for microbiota profiling were transported in dry ice and stored at − 80 °C until DNA extraction.

### DNA extraction

Total DNA was extracted from ~ 100 mg digesta, mucosa, and feed using the QIAamp DNA Stool Mini Kit (Qiagen, Hilden, Germany) as previously described [36], except that 2 mL prefilled PowerBead tubes (glass beads, 0.1 mm; Cat no. 13118-50, Qiagen) were used for the bead beating. To extract DNA from water samples, the frozen filter was allowed to soften on ice and rolled into a cylinder with the white filter membrane facing outward using two sets of sterile forceps. The filter was then inserted into an 8 mL sterile tube containing the double amount of ASL buffer and glass beads used in the prefilled PowerBead tubes. The tube was secured horizontally to a mixer mill (Retsch GmbH, Germany; model, MM 301) and shaken vigorously at the frequency of 30 Hz for 5 min (2.5 min, pause and invert the tube, 2.5 min). After shaking, the tube was centrifuged at 4000 g for 1 min, and 2.6 mL supernatant was collected and evenly aliquoted into two 1.5 mL Eppendorf tubes. The DNA was extracted from the supernatant aliquots and pooled afterward, following the protocol as previously described [36]. For quality control purposes, a companion “blank extraction” sample was added to each batch of sample DNA extraction by omitting the input material, whereas an additional mock sample (ZymoBIOMICS™, Zymo Research, California, USA; catalog no., D6300) was included for each DNA extraction kit as a positive control. The mock consists of 8 bacteria (*Pseudomonas aeruginosa*, *Escherichia coli*, *Salmonella enterica*, *Lactobacillus fermentum*, *Enterococcus faecalis*, *Staphylococcus aureus*, *Listeria monocytogenes*, *Bacillus subtilis*) and 2 yeasts (*Saccharomyces cerevisiae*, *Cryptococcus neoformans*).

### Library preparation and sequencing

The sequencing library was prepared using a two-step PCR protocol. In the first PCR, the V1-V2 hypervariable regions of the bacterial 16S rRNA gene were amplified using the primer set 27F (5′-AGA GTT TGA TCM TGG CTC AG-3′) and 338R (5′-GCW GCC WCC CGT AGG WGT-3′) [65]. The PCR was run in a total reaction volume of 25 μL containing 12.5 μL of Phusion® High-Fidelity PCR Master Mix (Thermo Scientific, CA, USA; catalog no., F531L), 10.5 μL molecular grade H_2_O, 1 μL DNA template, and 0.5 μL of each primer (10 μM). The amplification program was set as follows: initial denaturation at 98 °C for 3 min; 35 cycles of denaturation at 98 °C for 15 s, annealing decreasing from 63 to 53 °C in 10 cycles for 30 s followed by 25 cycles at 53 °C for 30 s, and extension at 72 °C for 30 s; followed by a final extension at 72 °C for 10 min. The PCR was run in duplicate incorporating negative PCR controls, which were generated by replacing the template DNA with molecular grade H_2_O. The duplicate PCR products were pooled and examined by a 1.5% agarose gel electrophoresis. Amplicons from the first PCR were cleaned using the Agencourt AMPure XP beads (Beckman Coulter, Indiana, USA; catalog no., A63881).

In the second PCR, sample barcodes and Illumina sequencing adapters were attached to the amplicons by dual indexing using the Nextera XT Index Kit (Illumina, California, USA; catalog no., FC-131-1096) [66]. PCR products from the second PCR were purified again using the AMPure XP beads. After the clean-up, representative libraries were selected and analyzed using the Agilent DNA 1000 Kit (Agilent Technologies, California, USA; catalog no., 5067-1505) to verify the library size. Cleaned libraries were quantified using the Invitrogen Qubit™ dsDNA HS Assay Kit (Thermo Fisher Scientific, California, USA; catalog no., Q32854), diluted to 4 nM in 10 mM Tris (pH 8.5) and finally pooled in an equal volume. Negative controls with library concentrations lower than 4 nM were pooled in equal volume directly. Due to the low diversity of amplicon library, 15% Illumina generated PhiX control (catalog no., FC-110-3001) was spiked in by combining 510 μL amplicon library with 90 μL PhiX control library. The pooled library was loaded onto the Miseq at 6 pM and sequenced using the Miseq Reagent Kit v3 (600-cycle) (Illumina; catalog no., MS-102-3003).

Due to technical challenges in obtaining high-quality PCR products for mucosa samples, the digesta samples were first amplified and sequenced. The PCR conditions for mucosa samples were optimized by diluting the DNA templates (1:5) to reduce the influence of PCR inhibitors. The mucosa samples were then sequenced in a second run together with feed and water samples. To assess potential batch effects between sequencing runs, 8 representative digesta samples were also sequenced in the second run to serve as technical replicates.

### Sequence data processing

The raw sequence data from each run were separately processed by the DADA2 (version 1.20) in R (version 4.1.1) [67] to infer amplicon sequence variants (ASVs) [68]. Specifically, the demultiplexed paired-ended reads were trimmed off the primer sequences (first 20 bps of forward reads and first 18 bps of reverse reads), truncated at the position where the median Phred quality score crashed (forward reads at position 290 bp and reverse reads at position 238 bp for the first run; forward reads at position 290 bp and reverse reads at position 248 bp for the second run) and filtered off low-quality reads. After the trimming and filtering, run-specific error rates were estimated, and the ASVs were inferred from each sample independently. The chimeras were removed using the “consensus” method after merging the forward and reverse reads. The resulting feature table and representative sequences from each run were imported into QIIME2 (version 2020.11) [69] and merged. The taxonomy was assigned by a scikit-learn naive Bayes machine-learning classifier [70], which was trained on the SILVA 132 99% OTUs [71] that were trimmed to only include V1–V2 regions of the 16S rRNA gene. Taxa identified as chloroplasts or mitochondria were excluded from the feature table. The feature table was conservatively filtered to remove ASVs that had no phylum-level taxonomic assignments or appeared in only one biological sample. Contaminating ASVs were identified and removed based on two suggested criteria: contaminants are often found in negative controls and inversely correlate with sample DNA concentration [72], which was quantified by qPCR as previously described [29]. The ASVs filtered from the feature table were also removed from the representative sequences, which were then inserted into a reference phylogenetic tree built on the SILVA 128 database using the SEPP [73]. The alpha-diversity indices were computed by rarefying the feature table at a subsampling depth of 10 532 sequences. To compare beta-diversity, we performed robust Aitchison PCA using the QIIME2 library DEICODE [74], which is a form of Aitchison distance that is robust to high levels of sparsity in the microbiome data via matrix completion. Samples with less than 1000 sequences and sequences with less than 10 total counts were excluded when running the robust Aitchison PCA. For downstream data visualization and statistical analyses, QIIME2 artifacts were imported into R using the qiime2R (version 0.99.35) package [75] and a phyloseq (version 1.38) [76] object was assembled. As the technical replicates showed strong batch effects between the sequencing runs, which could not be effectively removed by existing batch effect correction methods such as RUVSeq [77] and ComBat-seq [78], we performed the downstream data analysis independently for samples sequenced in different runs.

### Statistics

Differences in the alpha-diversity indices were compared by linear mixed-effects models using the R package afex (version 1.0-1) [79], which runs the lme4 [80] under the hood to fit mixed-effects models. Predictor variables in the models include the fixed effects Diet + Segment + Diet × Segment, and the random effects FishID + Tank. The homoscedasticity and normality of model residuals were visually assessed by inspecting diagnostic plots generated by the R package ggResidpanel (version 0.3.0) [81]. When necessary, data were log-transformed to meet the model assumptions. The statistical significance of fixed predictors was estimated by Type III *ANOVA* with Kenward–Roger’s approximation [82] of denominator degrees of freedom. When the interaction between the main effects was significant, conditional contrasts for the main effects were made using the R package emmeans (version 1.7.0) [83]. To compare differences in the beta-diversity, we performed the PERMANOVA [84] with 999 permutations in the PRIMER v7 (Primer-E Ltd., Plymouth, UK) using the same predictors included in the linear mixed-effects models. Terms with negative estimates for components of variation were sequentially removed from the model via term pooling, starting with the one showing the smallest mean squares. At each step, the model was reassessed whether more terms needed to be removed or not. Conditional contrasts for the main effects were constructed when their interaction was significant. Monte Carlo *p* values were computed as well when the unique permutations for the terms in the PERMANOVA were small (< 100). The homogeneity of multivariate dispersions among groups was visually assessed by boxplots and formally tested by the PERMDISP [85] with 999 permutations using the R package vegan (version 2.5–7) [86]. Per-feature tests for the association between specific microbial clade and sample metadata were done using the R package MaAsLin2 (version 1.8.0) [87]. The feature table was collapsed at the genus level and bacterial taxa of low prevalence (present in < 25% of samples) were excluded before running the association analysis. Predictor variables included in the association testing are fixed factors Diet + Segment + *foxp3* (qPCR) + *plin2* (qPCR), and the random effects FishID + Tank. Multiple comparisons were adjusted by the Holm [88] or Benjamini-Hochberg [89] method where applicable. Differences were regarded as significant for *p* < 0.05 or FDR-corrected *q* < 0.1.

## Supplementary Information

**Additional file 1. Table S1:** Contaminating ASVs removed from the feature table. The primary contaminating ASVs were classified as *Pseudomonas*, *Halomonas*, *Shewanella algae*, *Undibacterium*, *Bradyrhizobium*, *Ralstonia*, *Chitinophagaceae*, *Sediminibacterium*, *Curvibacter*, *Afipia*, and *Cutibacterium*.

**Additional file 2. Figures S1 to S3.** Supplementary figures.

## Data Availability

The raw 16S rRNA gene sequencing data are deposited at the NCBI SRA database under the BioProject PRJNA730696. The code for reproducing our results is available at the GitHub repository (https://github.com/yanxianl/Li_AqFl1-Microbiota_2021).
