# Supplementary material for: Consistent changes in the intestinal microbiota of Atlantic salmon fed insect meal diets
Source: Anim Microbiome. 2022 Jan 10;4:8. doi: 10.1186/s42523-021-00159-4 (PMC8750867; doi:10.1186/s42523-021-00159-4)
Supplement: Supplementary file 2 — Additional file 2. Figures S1 to S3. Supplementary figures. [file 42523_2021_159_MOESM2_ESM.pdf]

Consistent changes in the intestinal microbiota of Atlantic salmon fed insect meal diets

Yanxian Li[1*], Karina Gajardo[1], Alexander Jaramillo-Torres[1], Trond M. Kortner[1*], Åshild Krogdahl[1]

[1]Department of Paraclinical Sciences, Faculty of Veterinary Medicine, Norwegian University of Life Sciences, Oslo, Norway.

[*]Correspondence authors: yanxian.li@nmbu.no; trond.kortner@nmbu.no

Supplementary figures

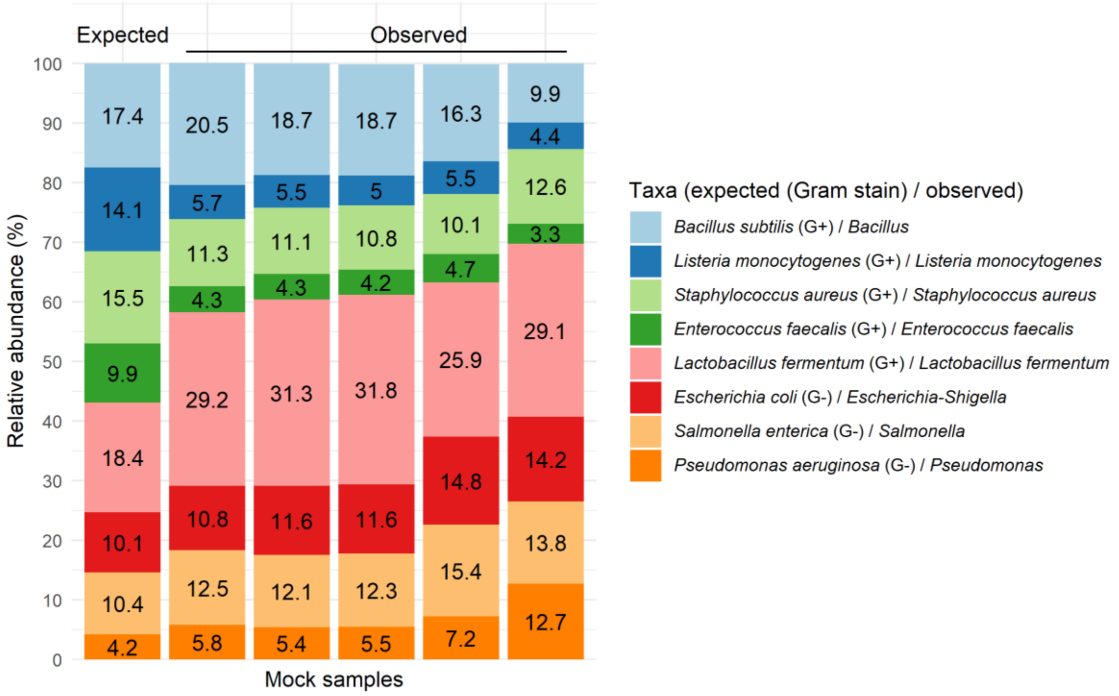

Figure S1. The expected and observed taxonomic composition of the ZymoBIOMICS mock standard. All the bacterial strains in the mock standard were correctly identified at the genus level with *E. faecalis*, *L. fermentum*, *L. monocytogenes,* and *S. aureus* further being assigned at the species level. At the genus level, the average correlation coefficient (Pearson's *r*) between the expected and observed taxonomic composition of the mock standard is 0.61.

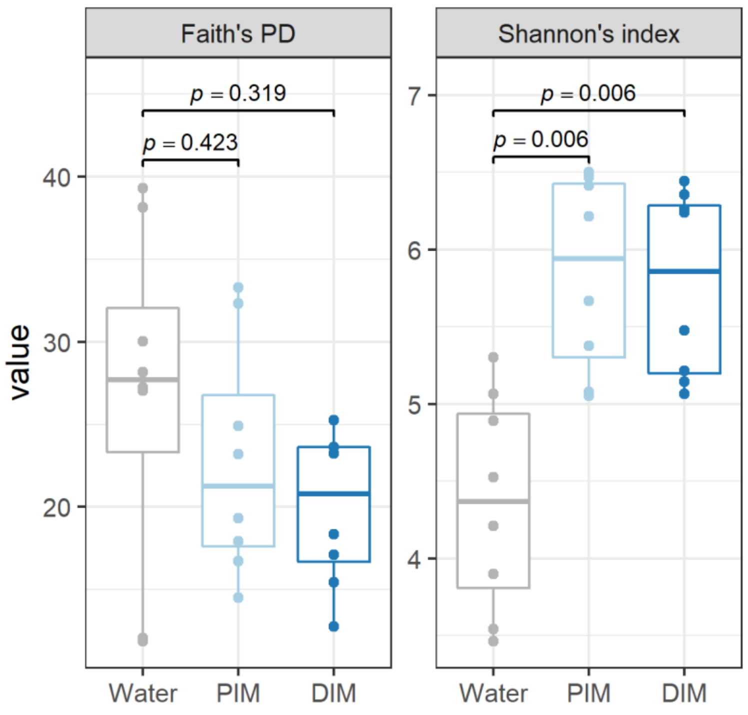

Figure S2. Comparison of alpha-diversity between paired water and intestinal mucosa samples. Note that alpha-diversity indices of intestinal mucosa samples from the same tank were aggregated before running paired *t*-test. Abbreviations: PIM, proximal intestine mucosa; DIM, distal intestine mucosa; PD, phylogenetic diversity.

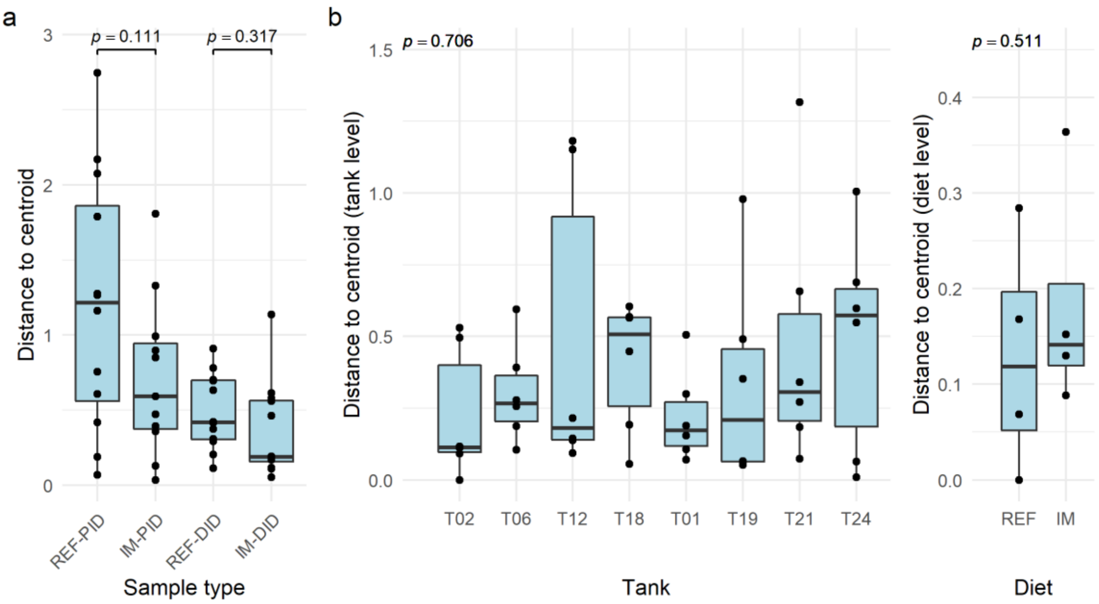

Figure S3. Tests for homogeneity of multivariate dispersions (PERMDISP) in digesta (a) and mucosa (b) samples. (a) The random effect, tank, was not included in the final PERMANOVA model. Therefore, we used individual fish as the statistical unit when running the PERDISP. (b) The PERDISP, however, was carried out at the tank and diet level for mucosa samples because the random effect, tank, was included in the final PERMANOVA model. Abbreviations: REF, reference diet; IM, insect meal diet; PID, proximal intestine digesta; DID, distal intestine digesta.
